# Correction: Modeling the initial phase of COVID-19 epidemic: The role of age and disease severity in the Basque Country, Spain

**DOI:** 10.1371/journal.pone.0314214

**Published:** 2024-11-14

**Authors:** Akhil Kumar Srivastav, Nico Stollenwerk, Joseba Bidaurrazaga Van-Dierdonck, Javier Mar, Oliver Ibarrondo, Maíra Aguiar

The first author’s name is spelled incorrectly. The correct name is: Akhil Kumar Srivastav. The correct citation is: Srivastav AK, Stollenwerk N, Bidaurrazaga Van-Dierdonck J, Mar J, Ibarrondo O, Aguiar M (2022) Modeling the initial phase of COVID-19 epidemic: The role of age and disease severity in the Basque Country, Spain. PLOS ONE 17(7): e0267772. https://doi.org/10.1371/journal.pone.0267772.
