# Supplementary material for: Modeling the initial phase of COVID-19 epidemic: The role of age and disease severity in the Basque Country, Spain
Source: PLoS One. 2022 Jul 13;17(7):e0267772. doi: 10.1371/journal.pone.0267772 (PMC9278753; doi:10.1371/journal.pone.0267772)
Supplement: S1 File — (PDF) [file pone.0267772.s001.pdf]

Supporting information

S1 Appendix. Computation of the basic reproduction number R_0: We find the basic reproduction number R_0 by following the next generation matrix method[40]. We consider $(E_1, A_1, H_1, E_2, A_2, H_2)$ for the vector $\mathcal{F}$ and $\mathcal{V}$ as follows:

$$\mathcal{F} = \begin{pmatrix} \beta S_1[\phi\{A_1 + \epsilon A_2\} + (H_1 + H_2)] \\ 0 \\ 0 \\ \beta S_1[\phi\{A_1 + \epsilon A_2\} + (H_1 + H_2)] \\ 0 \\ 0 \end{pmatrix}$$

and

$$\mathcal{V} = \begin{pmatrix} \eta_1 E_1 \\ -a\eta_1 E_1 + \alpha_1 A_1 \\ -(1-a)\eta_1 E_1 + \delta_1 H_1 + \alpha_2 H_1 \\ \eta_2 E_2 \\ -a\eta_2 E_2 + \alpha_3 A_2 \\ -(1-a)\eta_2 E_1 + \delta_2 H_2 + \alpha_4 H_2 \end{pmatrix},$$

F is the Jacobian matrix of $\mathcal{F}$ at E_0 hence

$$F = \begin{pmatrix} 0 & \beta_1\phi S_1^0 & \beta S_1^0 & 0 & \beta\epsilon\phi S_1^0 & \beta S_1^0 \\ 0 & 0 & 0 & 0 & 0 & 0 \\ 0 & 0 & 0 & 0 & 0 & 0 \\ 0 & \beta\phi S_2^0 & \beta S_2^0 & 0 & \beta\epsilon\phi S_2^0 & \beta_2 S_2^0 \\ 0 & 0 & 0 & 0 & 0 & 0 \\ 0 & 0 & 0 & 0 & 0 & 0 \end{pmatrix}$$

and V is the Jacobian matrix of $\mathcal{V}$ at E_0 hence

$$V = \begin{pmatrix} \eta_1 & 0 & 0 & 0 & 0 & 0 \\ -a\eta_1 & \alpha_1 & 0 & 0 & 0 & 0 \\ -(1-a)\eta_1 & 0 & (\delta_1 + \alpha_2) & 0 & 0 & 0 \\ 0 & 0 & 0 & \eta_2 & 0 & 0 \\ 0 & 0 & 0 & -a\eta_2 & \alpha_3 & 0 \\ 0 & 0 & 0 & -(1-a)\eta_2 & 0 & (\delta_2 + \alpha_4) \end{pmatrix}$$

Then calculated matrix FV^{-1} and simplifying, $|\lambda I - FV^{-1}| = 0$ gives the characteristics equation as follows:

$$a_1\lambda^2 - a_2\lambda - a_3 = 0,$$

Where $a_1 = 1, \quad a_2 = (a_{11} + a_{44}), \quad a_3 = a_{11}a_{44} - a_{14}a_{41}$ and

$$a_{11} = \beta S_1^0 \left\{ \frac{(1-a)}{(\delta_1 + \alpha_2)} + \frac{\phi a}{\alpha_1} \right\}, a_{14} = \beta S_1^0 \left\{ \frac{(1-a)}{(\delta_2 + \alpha_4)} + \frac{\phi \epsilon a}{\alpha_3} \right\},$$

$$a_{41} = \beta S_2^0 \left\{ \frac{(1-a)}{(\delta_1 + \alpha_2)} + \frac{\phi}{\alpha_1} \right\}, a_{44} = \beta S_2^0 \left\{ \frac{(1-a)}{(\delta_2 + \alpha_4)} + \frac{\phi \epsilon a}{\alpha_3} \right\},$$

So the basic reproduction number (R_0) is the positive root of the above quadratic and is given by:

$$R_0 = \beta S_1^0 \left\{ \frac{(1-a)}{(\delta_1 + \alpha_2)} + \frac{\phi a}{\alpha_1} \right\} + \beta S_2^0 \left\{ \frac{(1-a)}{(\delta_2 + \alpha_4)} + \frac{\phi \epsilon a}{\alpha_3} \right\}$$

S2 Appendix. Proof of theorem 1.2:

Proof: To prove the global stability of disease free equilibrium, we are using the theorem by Castillo-Chavez et. al. [48], and re-writing the equations for the exposed, infected and hospitalization compartments in (1) we get

$$\begin{pmatrix} E_1 \\ A_1 \\ H_1 \\ E_2 \\ A_2 \\ H_2 \end{pmatrix} = (F - V) \begin{pmatrix} E_1 \\ A_1 \\ H_1 \\ E_2 \\ A_2 \\ H_2 \end{pmatrix} - \begin{pmatrix} \beta S_1[\phi\{A_1 + \epsilon A_2\} + (H_1 + H_2)] \\ 0 \\ 0 \\ \beta S_2[\phi\{A_1 + \epsilon A_2\} + (H_1 + H_2)] \\ 0 \\ 0 \end{pmatrix}.$$

If all the eigenvalues of the matrix $F - V$ have negative real parts, the system is stable for $R_0 < 1$. So, By the comparison theorem, it follows that

$$({E_1}^0, {A_1}^0, {H_1}^0, {E_2}^0, {A_2}^0, {H_2}^0) \to (0, 0, 0, 0, 0, 0)$$

as $t \to \infty$. Then $({S_1}^0, {E_1}^0, {A_1}^0, {H_1}^0, {S_2}^0, {E_2}^0, {A_2}^0, {H_2}^0, R^0, D^0) \to E_0$ as $t \to \infty$. So, E_0 is globally asymptotically stable for $R_0 < 1$.

S3 Appendix. Stochastic Process of the SIRS Model:
SIRS Deterministic Model:

$$
\begin{aligned}
\frac{dS}{dt} &= -\beta SI + \alpha R \\
\frac{dI}{dt} &= \beta SI - \gamma I \\
\frac{dR}{dt} &= \gamma I - \alpha R
\end{aligned}
$$

Let us consider the case when one uninfected human is infected by a covid virus. In that case, the state change ΔX is denoted by $\Delta X = (-1, 1, 0)$ and its probability is given by

$$
\begin{aligned}
&\text{prob}(\Delta X_1, \Delta X_2, \Delta X_3) \\
&\qquad = \text{prob}((-1, 1, 0)|(X_1, X_2, X_3)) \\
&\qquad = P_1 = \beta X_1 X_2 \Delta t + O\Delta(t).
\end{aligned}
$$

One can easily determine the expectation change $E(\Delta X)$ and its covariance matrix $V(\Delta X)$ associated with ΔX by neglecting the terms higher than $O(\Delta t)$. The expectation of ΔX is given by

$$
\begin{aligned}
E(\Delta X) &= \sum_{i=1}^{3} P_i (\Delta X)_i \Delta t \\
&= \begin{pmatrix} -\beta X_1 X_2 + \alpha X_3 \\ \beta X_1 X_2 - \gamma X_2 \\ \gamma X_2 - \alpha X_3 \end{pmatrix} \Delta t \\
&= f(X_1, X_2, X_3)\Delta t.
\end{aligned}
$$

Here, it can be noted that the expectation vector and the function f are in the same form as those in the deterministic system. Since the covariance matrix is given by$V(\Delta X) = E((\Delta X)(\Delta X)^T) - E(\Delta X)E((\Delta X)^T)$ and $E(\Delta X)E((\Delta X)^T) = f(X)(f(X)^T)$, it can be approximated with diffusion matrix Ω times Δt by neglecting the term of $(\Delta t)^2$ such that

$$
\begin{aligned}
E((\Delta X)(\Delta X)^T) &= \sum_{i=1}^{3} P_i ((\Delta X)_i (\Delta X)_i^T) \Delta t \\
&= \begin{pmatrix} V_{11} & V_{12} & V_{13} \\ V_{21} & V_{22} & V_{23} \\ V_{31} & V_{32} & V_{33} \end{pmatrix} \cdot \Delta t = \Omega \cdot \Delta t,
\end{aligned}
$$

where the above diffusion matrix is symmetric, positive-definite and each component of this 3×3 diffusion matrix is given by

$$\begin{aligned}
V_{11} &= P_1 + P_3 = \beta X_1 X_2 + \alpha X_3,\ V_{12} = V_{21} = -P_1 = -\beta X_1 X_2, \\
V_{22} &= P_1 + P_2 = \beta X_1 X_2 + \gamma X_2 \\
V_{23} &= V_{32} = -P_2 = -\gamma X_2,\ V_{33} = P_2 + P_3 = \gamma X_2 + \alpha X_3
\end{aligned}$$

Following [45], we construct a matrix M such that $\Omega = MM^T$, where M is a 3×3 matrix given as

$$M = \begin{pmatrix} M_1^1 & 0 & M_3^1 \\ M_2^1 & M_2^2 & 0 \\ 0 & M_3^2 & M_3^3 \end{pmatrix},$$

where

$$M_1^1 = -\sqrt{P_1},\ M_3^1 = \sqrt{P_3},\ M_2^1 = \sqrt{P_1},\ M_2^2 = -\sqrt{P_2},\ M_3^2 = \sqrt{P_2},\ M_3^3 = -\sqrt{P_3},$$

Then, the Ito stochastic differential model has the form,

$$d(X(t)) = f(X_1, X_2, X_3)dt + M \cdot dW(t)$$

with initial condition

$$X(0) = (X_1(0), X_2(0), X_3(0))^T$$

and a Wiener process, are along with the standard deviation σ

$$W(t) = (W_1(t), W_2(t), W_3(t))^T.$$

In view of the above facts, we construct the stochastic differential equation model as follows,

$$\begin{aligned}
dS &= [-\beta SI + \alpha R]dt - \sqrt{\beta SI}dW_1 + \sqrt{\alpha R}dW_2, \\
dI &= [\beta SI - \gamma I]dt + \sqrt{\beta SI}dW_1 - \sqrt{\gamma I}dW_3 \\
dR &= [\gamma I - \alpha R]dt + \sqrt{\gamma I}dW_3 - \sqrt{\alpha R}dW_2
\end{aligned}$$

S4 Appendix. Stochastic Process of the proposed Deterministic Model 1: We follow above Method to construct the stochastic process: Let us consider the case when one uninfected human is infected by a covid virus. In that case, the state change ΔX is denoted by $\Delta X = (-1, 1, 0, 0, 0, 0, 0, 0, 0, 0)$ and its probability is given by

$$\begin{aligned}
&\text{prob}(\Delta X_1, \Delta X_2, \Delta X_3, \Delta X_4, \Delta X_5, \Delta X_6, \Delta X_7, \Delta X_8, \Delta X_9, \Delta X_{10}) \\
&\qquad = \text{prob}((-1, 1, 0, 0, 0, 0, 0, 0, 0, 0)|(X_1, X_2, X_3, X_4, X_5, X_6, X_7, X_8, X_9, X_{10})) \\
&\qquad = P_1 = \beta X_1 X_2 \Delta t + O\Delta(t).
\end{aligned}$$

One can easily determine the expectation change $E(\Delta X)$ and its covariance matrix $V(\Delta X)$ associated with ΔX by neglecting the terms higher than $O(\Delta t)$. The expectation of ΔX is given by

$$\begin{aligned}
E(\Delta X) &= \sum_{i=1}^{13} P_i(\Delta X)_i \Delta t \\
&= \begin{pmatrix}
-\beta X_1[\phi\{X_3 + \epsilon X_7\} + (X_4 + X_8)] \\
\beta X_1[\phi\{X_3 + \epsilon X_7\} + (X_4 + X_8)] - \eta_1 X_2 \\
a\eta_1 X_2 - \alpha_1 X_3 \\
(1-a)\eta_1 X_2 - \delta_1 X_4 - \alpha_2 X_4. \\
-\beta X_5[\phi\{X_3 + \epsilon X_7\} + (X_4 + X_8)] \\
\beta X_5[\phi\{X_3 + \epsilon X_7\} + (X_4 + X_8)] - \eta_2 X_6 \\
a\eta_2 X_6 - \alpha_3 X_7 \\
(1-a)\eta_2 X_6 - \delta_2 X_8 - \alpha_4 X_8. \\
\alpha_1 X_3 + \alpha_2 X_4 + \alpha_3 X_7 + \alpha_4 X_8 \\
\delta_1 X_4 + \delta_2 X_8
\end{pmatrix} \Delta t \\
&= f(X_1, X_2, X_3, X_4, X_5, X_6, X_7, X_8, X_9, X_{10})\Delta t.
\end{aligned}$$

Here, it can be noted that the expectation vector and the function f are in the same form as those in deterministic system (1). Since the covariance matrix $V(\Delta X) = E((\Delta X)(\Delta X)^T) - E(\Delta X)E((\Delta X)^T)$ and $E(\Delta X)E((\Delta X)^T) = f(X)(f(X)^T)$, it can be approximated with diffusion matrix Ω times Δt by neglecting the term of $(\Delta t)^2$

such that

$$
\begin{aligned}
E((\Delta X)(\Delta X)^T) &= \sum_{i=1}^{10} P_i((\Delta X)_i(\Delta X)_i^T)\Delta t \\
&= \begin{pmatrix}
V_{11} & V_{12} & 0 & 0 & 0 & 0 & 0 & 0 & 0 & 0 \\
V_{21} & V_{22} & V_{23} & V_{24} & 0 & 0 & 0 & 0 & 0 & 0 \\
0 & V_{32} & V_{33} & 0 & 0 & 0 & 0 & 0 & V_{39} & 0 \\
0 & V_{42} & 0 & V_{44} & 0 & 0 & 0 & 0 & V_{49} & V_{4,10} \\
0 & 0 & 0 & 0 & V_{55} & V_{56} & 0 & 0 & 0 & 0 \\
0 & 0 & 0 & 0 & V_{65} & V_{66} & V_{67} & V_{68} & 0 & 0 \\
0 & 0 & 0 & 0 & 0 & V_{76} & V_{77} & 0 & V_{79} & 0 \\
0 & 0 & 0 & 0 & 0 & V_{86} & 0 & V_{88} & V_{89} & V_{8,10} \\
0 & 0 & V_{93} & V_{94} & 0 & 0 & V_{97} & V_{98} & V_{99} & 0 \\
0 & 0 & 0 & V_{10,4} & 0 & 0 & 0 & V_{10,8} & 0 & V_{10,10}
\end{pmatrix} \cdot \Delta t \\
&= \Omega \cdot \Delta t,
\end{aligned}
$$

where the above diffusion matrix is symmetric, positive-definite and each component of this 10×10 diffusion matrix are given by

$$
\begin{aligned}
V_{11} &= P_1 = \beta X_1[\phi\{X_3 + \epsilon X_7\} + (X_4 + X_8)], \\
V_{12} &= V_{21} = -P_1 = -\beta X_1[\phi\{X_3 + \epsilon X_7\} + (X_4 + X_8)], \\
V_{22} &= P_1 + P_2 + P_3 = \beta X_1[\phi\{X_3 + \epsilon X_7\} + (X_4 + X_8)] + a\eta_1 X_2 + (1-a)\eta_1 X_2 \\
V_{23} &= V_{32} = -P_2 = -a\eta_1 X_2,\ V_{24} = V_{42} = -P_3 = -(1-a)\eta_1 X_2, \\
V_{33} &= P_2 + P_4 = a\eta_1 X_2 + \alpha_1 X_3,\ V_{39} = V_{93} = -P_4 = -\alpha_1 X_3, \\
V_{44} &= P_3 + P_5 + P_6 = (1-a)\eta_1 X_2 + \delta_1 X_4 + \alpha_2 X_4, \\
V_{4,10} &= V_{10,4} = -P_5 = -\delta_1 X_4,\ V_{55} = P_7 = \beta X_5[\phi\{X_3 + \epsilon X_7\} + (X_4 + X_8)], \\
V_{56} &= V_{65} = -P_7 = -\beta X_5[\phi\{X_3 + \epsilon X_7\} + (X_4 + X_8)], \\
V_{66} &= P_7 + P_8 + P_9 = \beta X_5[\phi\{X_3 + \epsilon X_7\} + (X_4 + X_8)] + a\eta_2 X_6 + (1-a)\eta_2 X_6, \\
V_{67} &= V_{76} = -P_8 = -a\eta_2 X_6, V_{68} = V_{86} = -P_9 = -(1-a)\eta_2 X_6, \\
V_{77} &= P_8 + P_{10} = a\eta_2 X_6 + \alpha_3 X_7,\ V_{79} = V_{97} = -P_{10} = \alpha_3 X_7 \\
V_{88} &= P_9 + P_{11} + P_{12} = (1-a)\eta_2 X_6 + \delta_2 X_8 + \alpha_4 X_8, \\
V_{89} &= V_{98} = -P_{12} = -\alpha_4 X_8,\ V_{8,10} = V_{10,8} = -P_{11} = \delta_2 X_8, \\
V_{99} &= P_4 + P_6 + P_{10} + P_{12} = \alpha_1 X_3 + \alpha_2 X_4 + \alpha_3 X_7 + \alpha_4 X_8, \\
V_{10,10} &= P_5 + P_{11} = \delta_1 X_4 + \delta_2 X_8.
\end{aligned}
$$

Following [45], we construct a matrix M such that $\Omega = MM^T$, where M is a 10×12

matrix given as

$$M = \begin{pmatrix} M_{11} & 0 & 0 & 0 & 0 & 0 & 0 & 0 & 0 & 0 & 0 & 0 \\ M_{21} & M_{22} & M_{23} & 0 & 0 & 0 & 0 & 0 & 0 & 0 & 0 & 0 \\ 0 & M_{32} & 0 & M_{34} & 0 & 0 & 0 & 0 & 0 & 0 & 0 & 0 \\ 0 & 0 & M_{43} & 0 & M_{45} & M_{46} & 0 & 0 & 0 & 0 & 0 & 0 \\ 0 & 0 & 0 & 0 & 0 & 0 & M_{57} & 0 & 0 & 0 & 0 & 0 \\ 0 & 0 & 0 & 0 & 0 & 0 & M_{67} & M_{68} & M_{69} & 0 & 0 & 0 \\ 0 & 0 & 0 & 0 & 0 & 0 & 0 & M_{78} & 0 & M_{7,10} & 0 & 0 \\ 0 & 0 & 0 & 0 & 0 & 0 & 0 & 0 & M_{89} & 0 & M_{8,11} & M_{8,12} \\ 0 & 0 & 0 & M_{94} & 0 & M_{96} & 0 & 0 & 0 & M_{9,10} & 0 & M_{9,12} \\ 0 & 0 & 0 & 0 & M_{10,5} & 0 & 0 & 0 & 0 & 0 & M_{10,11} & 0 \end{pmatrix},$$

where

$$\begin{aligned} &M_{11} = -\sqrt{P_1},\ M_{21} = \sqrt{P_1},\ M_{22} = -\sqrt{P_2},\ M_{32} = \sqrt{P_2},\ M_{23} = -\sqrt{P_3},\ M_{43} = \sqrt{P_3}, \\ &M_{34} = -\sqrt{P_4},\ M_{94} = \sqrt{P_4},\ M_{45} = -\sqrt{P_5},\ M_{10,5} = \sqrt{P_5},\ M_{46} = -\sqrt{P_6},\ M_{96} = \sqrt{P_6}, \\ &M_{57} = -\sqrt{P_7},\ M_{67} = \sqrt{P_7},\ M_{68} = -\sqrt{P_8},\ M_{78} = \sqrt{P_8},\ M_{69} = -\sqrt{P_9},\ M_{89} = \sqrt{P_9}, \\ &M_{7,10} = -\sqrt{P_{10}},\ M_{9,10} = \sqrt{P_{10}},\ M_{8,11} = -\sqrt{P_{11}},\ M_{10,11} = \sqrt{P_{11}}, \\ &M_{8,12} = -\sqrt{P_{12}},\ M_{9,12} = \sqrt{P_{12}}, \end{aligned}$$

Then, the Ito stochastic differential model has the form,

$$d(X(t)) = f(X_1, X_2, X_3, X_4, X_5, X_6, X_7, X_8, X_9, X_{10})dt + M \cdot dW(t)$$

with initial condition

$$X(0) = (X_1(0), X_2(0), X_3(0), X_4(0), X_5(0), X_6(0), X_7(0), X_8(0), X_9(0), X_{10}(0))^T$$

and a Wiener process, are along with the standard deviation σ

$$W(t) \;=\; (W_1(t), W_2(t), W_3(t), W_4(t), W_5(t), W_6(t), W_7(t), W_8(t), W_9(t), W_{10}(t), W_{11}(t), W_{12}(t))^T$$

In view of the above facts, we construct the stochastic differential equation model as follows,

$$
\begin{aligned}
dS_1 &= (-\beta S_1[\phi\{A_1+\epsilon A_2\}+(H_1+H_2)])dt - \sqrt{\beta S_1[\phi\{A_1+\epsilon A_2\}+(H_1+H_2)]}dW_1,\\
dE_1 &= [\beta S_1[\phi\{A_1+\epsilon A_2\}+(H_1+H_2)] - a\eta_1 E_1 - (1-a)\eta_1 E_1]dt\\
&\quad + \sqrt{\beta S_1[\phi\{A_1+\epsilon A_2\}+(H_1+H_2)}dW_1 - \sqrt{a\eta_1 E_1}dW_2 - \sqrt{(1-a)\eta_1 E_1}dW_3\\
dA_1 &= [a\eta_1 E_1 - \alpha_1 A_1]dt + \sqrt{a\eta_1 E_1}dW_2 - \sqrt{\alpha_1 A_1}dW_4\\
dH_1 &= [(1-a)\eta_1 E_1 - \delta_1 H_1 - \alpha_2 H_1]dt + \sqrt{(1-a)\eta_1 E_1}dW_3 - \sqrt{\delta_1 H_1}dW_5 - \sqrt{\alpha_2 H_1}dW_6\\
dS_2 &= (-\beta S_2[\phi\{A_1+\epsilon A_2\}+(H_1+H_2)])dt - \sqrt{\beta S_2[\phi\{A_1+\epsilon A_2\}+(H_1+H_2)]}dW_7,\\
dE_2 &= [\beta S_2[\phi\{A_1+\epsilon A_2\}+(H_1+H_2)] - a\eta_2 E_2 - (1-a)\eta_2 E_2]dt\\
&\quad + \sqrt{\beta S_2[\phi\{A_1+\epsilon A_2\}+(H_1+H_2)}dW_7 - \sqrt{a\eta_2 E_2}dW_8 - \sqrt{(1-a)\eta_2 E_2}dW_9\\
dA_2 &= [a\eta_2 E_2 - \alpha_3 A_2]dt + \sqrt{a\eta_2 E_2}dW_8 - \sqrt{\alpha_3 A_2}dW_{10}\\
dH_2 &= [(1-a)\eta_2 E_2 - \delta_2 H_2 - \alpha_4 H_4]dt + \sqrt{(1-a)\eta_2 E_2}dW_9 - \sqrt{\delta_2 H_2}dW_{11} - \sqrt{\alpha_4 H_2}dW_{12}\\
dR &= [\alpha_1 A_1 + \alpha_2 H_1 + \alpha_3 A_2 + \alpha_4 H_2]dt + \sqrt{\alpha_1 A_1}dW_4 + \sqrt{\alpha_2 H_1}dW_6 + \sqrt{\alpha_3 A_2}dW_{10}\\
&\quad + \sqrt{\alpha_4 H_2}dW_{12}\\
dD &= [\delta_1 H_1 + \delta_2 H_2]dt + \sqrt{\delta_1 H_1}dW_5 + \sqrt{\delta_2 H_2}dW_{11}
\end{aligned}
$$
