# Supplementary material for: Modeling the initial phase of COVID-19 epidemic: The role of age and disease severity in the Basque Country, Spain
Source: PLoS One. 2022 Jul 13;17(7):e0267772. doi: 10.1371/journal.pone.0267772 (PMC9278753; doi:10.1371/journal.pone.0267772)
Supplement: Attachment — Submitted filename: reviewers_June13_Aguiar.pdf [file pone.0267772.s002.pdf]

Dr. Maíra Aguiar
Mathematical and Theoretical Biology
(MTB) Group Leader
BCAM - Basque Center for Applied Mathematics
maguiar@bcamath.org | www.maira-aguiar.eu

June 13, 2022

Dear Reviewers of PLOS One,

Thank you very much for giving us an opportunity to revise our manuscript. We really appreciate the useful comments made by the reviewers to improve our manuscript. We have revised the manuscript and implemented all the suggestions. Below, please find our response to each of the comments. The updates are implemented in the revised manuscript highlighted in blue.

Looking forward to hearing from you

Sincerely yours,

Maíra Aguiar
(On behalf of the authors)

Reviewer 1:

The manuscript deals with a deterministic stage structured model along with corresponding stochastic extension. Here authors have chosen SEIHR model for both young and old populations and derived their interaction pattern. Interestingly they found that the younger population may be able to affect the disease spread which eventually cause fatal impact to older population. This is a preliminary study on initial phase of the pandemic. The work is reasonably good and may be accepted for publication. .

There are a few corrections **(mostly typos)** which authors should take cere of. For author's help I have mentioned a few.

1. In abstract they mentioned 'younger than 60' but in line 400 it is 'younger than 40' for young population.

2. **Response:** Thank you for bringing this to our attention. We have fitted data with model solutions for 'younger than 60' and 'younger than 40' for both, young and old populations, using both, normalized and non-normalized data. It was shown that 'younger than 60' has a better fitting than 'younger than 40'. As a result, 'younger than 60' was chosen to continue our analysis, assuming the threshold for “better” immunity level in respect to developing severe disease than

the older population which are more likely to die. The main results of this study are in respect to the 'younger than 60' population and that is the reason for us to mention 'younger than 60' in the abstract rather than 'younger than 40'.

3. In section 1.1 Ei etc should be defined as E1, E2 etc for better understanding as there are only two classes.

Response: Thank you for suggestion. We have updated the manuscript accordingly.

4. Theorem 1.1 and 1.2, components of E0 should be 10. Pl remove extra zeros.

Response: Thank you for pointing this out. We have corrected it in the manuscript.

5. Line 140, replace prove by state.

Response: Thank you for the suggestion. We have corrected it in the manuscript.

6. Expression after line 150, correct the term as $X_8(t)$

Response: Thank you for pointing this out. We have corrected it in the manuscript.

7. Line 169, write 600,000 instead of 600 thousand

Response: Thank you for suggestion. We have updated the manuscript accordingly.

8. Line 197, it should be pharmaceutical not non-pharmaceutical

Response: Thank you for pointing this out. Kindly note that here we are talking about non-pharmaceutical intervention measures, e.g social distancing and use of masks. We have made that clearer in the manuscript.

9. In caption of figure 3 and 6, replace left by right and right by left.

Response: Thank you for pointing this out. We have corrected it in the manuscript.

10. Caption Fig 12, write "The following .....". Also check expression of $\beta(t)$, it is incorrect.

Response: Thank you for pointing this out. We have corrected \beta(t) in the revised manuscript. For more information about this function, please refer to reference [1] below, which is cited in the manuscript.

[1] Aguiar, M., Mill ́an Ortuondo, E., Bidaurrazaga Van Dierdonck, J., Mar, J., Stollenwek, N.: Modelling COVID 19 in the Basque Country from introduction to control measure response. Sci. Rep. 10, 17306 (2020), doi:10.1038/s41598-020-74386

11. Line 386 ans 387: SIRS in place of SIR

Response: Thank you for pointing this out. We have updated the manuscript accordingly.

12. Expression before line 390: O(\Delta t) in place of O(t).

Response: Thank you for pointing this out. We have corrected the manuscript accordingly.

13. Line 405, \sigma seems undefined. Pls check.

Response: Thank you for pointing this out. We have defined \sigma in the revised manuscript. For more information about this function please refer to reference [1] above, which is cited in the manuscript.

Reviewer #2:

This manuscript deals with modeling COVID-19. The mathematical model is formulated by dividing the whole population under consideration into two different age groups, namely, older and younger keeping in view that the rates of transmission, hospitalization and recovery may vary for individuals in these two groups. The basic reproduction number (R0) is computed. The key parameters of the proposed model are estimated using nonlinear least square method based on data from the Basque Country, Spain. Sensitivity analysis is performed to see the impact of different parameters related to infectivity, recovery rates etc. The proposed model is extended to stochastic model and simulation results of both deterministic and stochastic models are compared. Overall manuscript is well written and mathematically sound. However, authors are requested to take a note of the following minor points and should make the necessary changes in the final manuscript:

1. Line no 202: "is" should be changed to "are".
Response: Thank you for suggestion. We have corrected it in the manuscript.
2. Caption of Figure 6: left hand side is deterministic and right hand side is stochastic.
Response: Thank you for pointing this out. We have corrected it in the manuscript.
3. Caption of Figure 11: "In a-c)" should be "In (a) and (c)". Similarly, "b-d)" should be "(b) and (d)".
Response: Thank you for suggestion. We have updated the manuscript accordingly.
4. Caption of Figure 12: "the following" should be "following". Check the control function included in this caption. x(t) is appearing with positive and negative signs.
Response: Thank you for pointing this out. We have defined \sigma in the revised manuscript. For more information about this function please refer to reference [1] above, which is cited in the manuscript.
5. Line 365 and 273: "dynamic" seems more appropriate in place of "dynamical".
Response: Thank you for suggestion. We have updated the manuscript accordingly.
